# Filamentous gram-negative bacteria masquerading as actinomycetes in infectious endophthalmitis: a review of three cases

**DOI:** 10.1186/s12348-018-0157-4

**Published:** 2018-10-11

**Authors:** Joveeta Joseph, Savitri Sharma, Vivek Pravin Dave

**Affiliations:** 1 Jhaveri Microbiology Centre, Brien Holden Eye Research Centre, L. V. Prasad Eye Institute, Hyderabad, Telangana 500034 India; 2 Smt. Kanuri Santhamma Centre for Vitreo-Retinal Diseases, L. V. Prasad Eye Institute, Hyderabad, India

**Keywords:** Actinomycetes, Masquerade, Endophthalmitis, Gram-negative bacteria

## Abstract

**Background:**

To report microbiological diagnostic dilemma posed by observation of unusual morphology of bacteria in the vitreous sample of a series of three cases of bacterial endophthalmitis.

**Results:**

A non-comparative, descriptive case series is described. All three cases presented to the retina-vitreous clinic with a clinical diagnosis of acute endophthalmitis between January and April 2018. Two patients had a past history of cataract surgery, and one had antecedent trauma within 1–2 days of presentation. As per the institutional protocol, patients underwent pars plana vitrectomy with intraocular antibiotics (vancomycin and ceftazidime) and microbiological investigation of the vitreous sample. Microscopic visualization of the stained vitreous fluid revealed the presence of filamentous organisms suggestive of *Actinomycetales*. However, the culture showed growth of gram-negative bacilli (*Pseudomonas aeruginosa*, *Klebsiella oxytoca*, *Morganella morganii*) which were identified by ViTEK 2 compact system and biochemical tests. Though a combination antibiotic treatment of vancomycin and ceftazidime was given in all cases in view of the short history, the antibiotic susceptibility testing showed multi-drug resistance pattern in two out of three cases leading to unfavorable clinical outcome.

**Conclusions:**

Gram-negative bacilli can develop abnormal morphology due to stress or sub-inhibitory antibiotic exposure, and it is important for ocular microbiologists and pathologists to be aware of this phenomenon to avoid misinterpretation that may lead to inappropriate treatment.

## Background

Infectious endophthalmitis remains one of the most challenging and devastating complications caused by an array of organisms and can quite possibly lead to devastating outcomes in the form of either permanent blindness or loss of the eyeball itself [1]. Differentiation of the underlying etiology might be difficult due to an overlap in clinical presentations, and ultrasonographic features may be equivocal. Often, there is one or more than one underlying etiology responsible for the patient’s clinical presentation. Hence, prompt identification of pathogens from clinical samples is fundamental to an appropriate and timely treatment that may go a long way in prevention of serious complications [1, 2]. Direct microscopic identification of a pathogen by its morphological features on staining forms the basis of diagnosis in most clinical set ups for guiding initial treatment [3]. The successful identification of the infectious disease pathology requires the proper grading of the inflammatory response, knowledge of associated pathogens, and in some cases use of special stains for characterization of the specific pathogen. However, bacteria may undergo several morphological changes when stressed with a host immune response and antibiotic exposure [4, 5], and these changes may potentially lead to misinterpretation, as it may not be routinely encountered.

Filamentation is a common response in which bacteria replicate but incompletely divide, leading to long slender chains [6] that resemble fungal hyphae or actinomycetes, a group of thin, gram-positive, beaded, branching bacteria placed in the order *Actinomycetales*. The members of actinomycetes share many overlapping morphologic features; however, antibiotic agents against them and the need for surgical intervention as part of the treatment plan vary greatly. Hence, the identification of microorganisms in vitreous fluids is a critical step in the diagnostic process and ultimately in patient care [3]. Herein, we report three patients with a diagnosis of acute infectious endophthalmitis that showed presence of filamentous bacteria mimicking *Actinomycetales* under microscopic examination of the vitreous fluids.

## Methods

The three cases reported here were among the endophthalmitis patients seen at L. V. Prasad Eye Institute, Hyderabad, between January 2018 and April 2018. All patients were initially empirically treated with intravitreal vancomycin (1 mg/0.1 ml) and ceftazidime (2.25 mg/0.1 ml) after collecting the vitreous sample, which was sent to the microbiology laboratory. Vitreous samples were taken from the anterior and mid vitreous using 23G or 25G trocar cannula set on the Constellation vitrectomy system (Alcon Laboratories, Fort Worth, TX).The sample was taken as a vitreous biopsy using a 2 cc syringe attached to a 23G vitreous cutter. Additional intravitreal antibiotic injections were given to the patient in the follow-up of the primary procedure based on the culture and sensitivity report. If no response was noted, a repeat intravitreal injection or a pars plana vitrectomy was done after 48–72 h depending on the corneal clarity. The outcome at the last visit was evaluated in terms of anatomic and functional outcome. A favorable anatomic outcome was defined as preservation of the globe, absence of hypotony, attached retina, and absence of active inflammation at the last visit. A functional success was defined as an attached retina with a vision of ≥ 20/400 at the last visit. Microbiological processing was immediate and involved direct microscopic examination (Calcofluor white, Gram, Giemsa stains) and culture on appropriate bacterial (aerobic and anaerobic) and fungal media as mentioned earlier [7]. Whenever there was a suspicion of presence of actinomycetes (i.e., presence of gram-positive, thin, beaded, filamentous bacilli, with or without branching), the sample was also processed for modified Ziehl-Neelsen stain using 1% H_2_SO_4_. The culture isolates were further processed for identification by biochemical tests and VITEK 2 compact system (bioMérieux, France) and antibiotic susceptibility testing by determination of minimum inhibitory concentration (MIC) using VITEK 2 compact system and E test (bioMérieux, France).

## Results

### Case 1

**Fig. 1:**
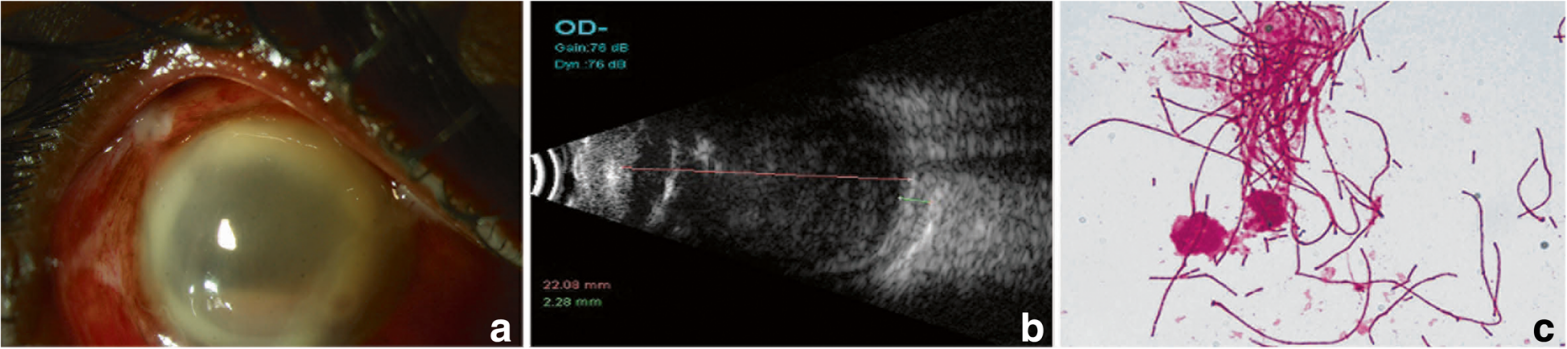
Case 1: **a** Slit lamp biomicroscopy of the anterior segment showed corneal edema with ring infiltrate and pinkish hypopyon with conjunctival chemosis. **b** B-scan ultrasonography showed multiple hyperechoic areas with attached retina with thickening of the choroid in the same case. **c** Gram stain of the vitreous biopsy showed filamentous gram-negative bacilli with pseudobranching (× 100) identified as *Pseudomonas aeruginosa* on culture

A 40-year-old man presented with redness, pain, and decreased vision in the right eye 1 day following cataract surgery done elsewhere. The patient had received intravitreal antibiotics (ceftazidime and vancomycin) prior to referral to us. His corrected distance visual acuity (CDVA) was light perception with accurate projection of rays. Slit lamp biomicroscopy of the anterior segment showed cloudy cornea with ring infiltrate and pinkish hypopyon along with congested, chemosed conjunctiva (Fig. 1a). B-scan ultrasonography showed multiple hyperechoic areas with the attached retina (Fig. 1b). Anterior chamber wash, with intravitreal imipenum (50 mg/0.1 mL), ceftazidime (2.25 mg/0.1 mL), and dexamethasone (400 μg/0.1 ml), was given. There was a delay in sending the sample to the microbiology laboratory by 16 h. Gram stain of the vitreous biopsy showed filamentous very long gram-negative bacilli, with doubtful branching (Fig. 1c) which was non-acid fast. The initial impression was that of actinomycetes although the gram-negative staining was contradictory. The next day, culture showed growth of greenish-gray moist colonies, which was identified as *Pseudomonas aeruginosa*. The organism was resistant to tobramycin, tigecycline, chloramphenicol, gentamicin, and moxifloxacin and was susceptible to colistin, ciprofloxacin, and ceftazidime as shown in Table 1. Systemic treatment included oral ciprofolxacin (750 mg BD) and oral prednisolone (4 mg/0.1 ml). Lack of clinical improvement necessitated intravitreal injections of ceftazidime and triamcinilone twice over. Once the exudates cleared, the patient was given systemic steroids with one dose of intravitreal dexamethasone (400 μg/0.1 mL). The patient improved symptomatically with no improvement in vision, and on the last follow-up at 10 days, his corneal lesions were stable and tectonically the eye appeared stable. AC appears well formed while the rest of the anterior segment details were not clear.

**Table 1:** Case-wise isolated gram-negative bacteria and their antibiotic susceptibility in micrograms per microliter concentration and reported as per CLSI 2017

Antibiotic tested	*Pseudomonas aeruginosa* Case 1	*Klebsiella oxytoca* Case 2	*Morganella morganii* Case 3
Piperacillin/tazobactam	16 (S)	≤ 4 (S)	≤ 4 (S)
Cefepime	4 (S)	≤ 1 (S)	8 (I)
Imipenem	2 (S)	0.5 (S)	4 (R)
Meropenem	1 (S)	≤ 0.25 (S)	0.5 (S)
Amikacin	8 (S)	≤ 2 (S)	16 (S)
Gentamicin	≥ 16 (R)	≤ 1 (S)	2 (S)
Ciprofloxacin	≤ 0.25 (S)	≤ 0.25 (S)	≥ 4 (R)
Tigecycline	≥ 8 (R)	≤ 0.25 (S)	4 (R)
Colistin	≥ 0.5 (S)	≤ 0.5 (S)	≥ 16 (R)
Trimthoprim/sulfamethoxazole	ND	≤ 20 (S)	≥ 20 (S)
Ceftazidime	12 (I)	0.5 (S)	16 (R)
Gatifloxacin	3 (I)	0.47 (S)	> 32 (R)
Ofloxacin	3 (I)	0.1 (S)	> 32 (R)
Moxifloxacin	2 (R)	0.09 (S)	> 32 (R)
Chloramphenicol	> 256 (R)	2 (S)	16 (R)
Tobramycin	> 256 (R)	1 (S)	64 (R)

*S* sensitive, *R* resistant, *I* intermediate, *ND* not done

### Case 2

**Fig. 2:**
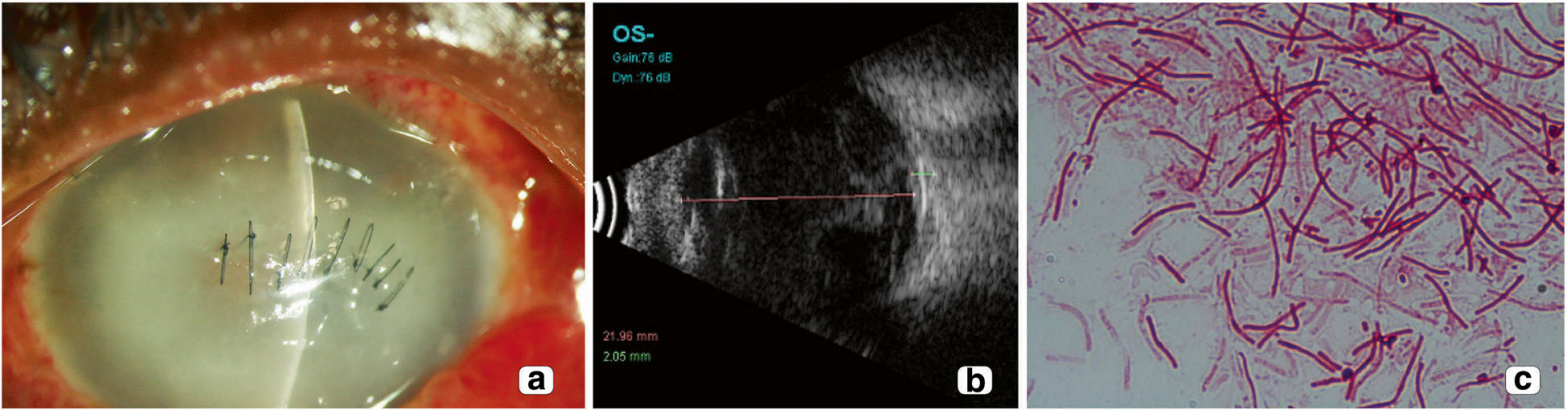
Case 2: **a** Slit lamp biomicroscopy of the anterior segment showed corneal edema, repaired corneal tear with ring infiltrate, and pinkish hypopyon along with conjunctival hyperemia of case 2. **b** B-scan ultrasonography showed multiple hyperechoic areas with attached retina of the same case. **c** Gram stain of the vitreous biopsy showed filamentous gram-negative bacilli with pseudobranching (× 100) identified as *Klebsiella oxytoca* on culture

A 44-year-old male presented with reduced vision in the left eye since a few hours. There was a history of trauma to the left eye with a stone a few hours back. At presentation, the vision in the right was 6/6, N6 and the clinical examination was normal. The left eye vision was recorded as hand motions with a central corneal tear, iris proplapse, 1 mm hypopyon, and a total traumatic cataract (Fig. 2a). A B-scan ultrasound to rule out a shallow retinal detachment which could not be assessed due to corneal opacity causing media haze was performed, and it showed attached retina with moderate intensity echoes (Fig. 2b). A provisional diagnosis of open globe injury with traumatic endophthalmitis was made, and the patient underwent left eye corneal tear repair, lensectomy, vitrectomy, and intraocular antibiotic injection. The vitreous sample however was kept at room temperature in the operating room overnight before being sent to the microbiology laboratory for processing. Gram stain of the vitreous biopsy showed long, thick gram-negative filamentous bacilli (Fig. 2c) giving an impression of actinomycete. However, in culture *Klebsiella oxytoca* was grown. The organism was sensitive to all antibiotics tested except ampicillin (Table 1). Over the next 1 week, the patient underwent a repeat intraocular antibiotic injection (ceftazidime and vancomycin) and an endoscopic vitrectomy due to the presence of significant retinal exudates possibly due to persistent infection not responding to treatment. At the last visit, 10 days post presentation, the vision was PL PR inaccurate with a repaired corneal tear and aphakia. The retina was attached on B-scan.

### Case 3

**Fig. 3:**
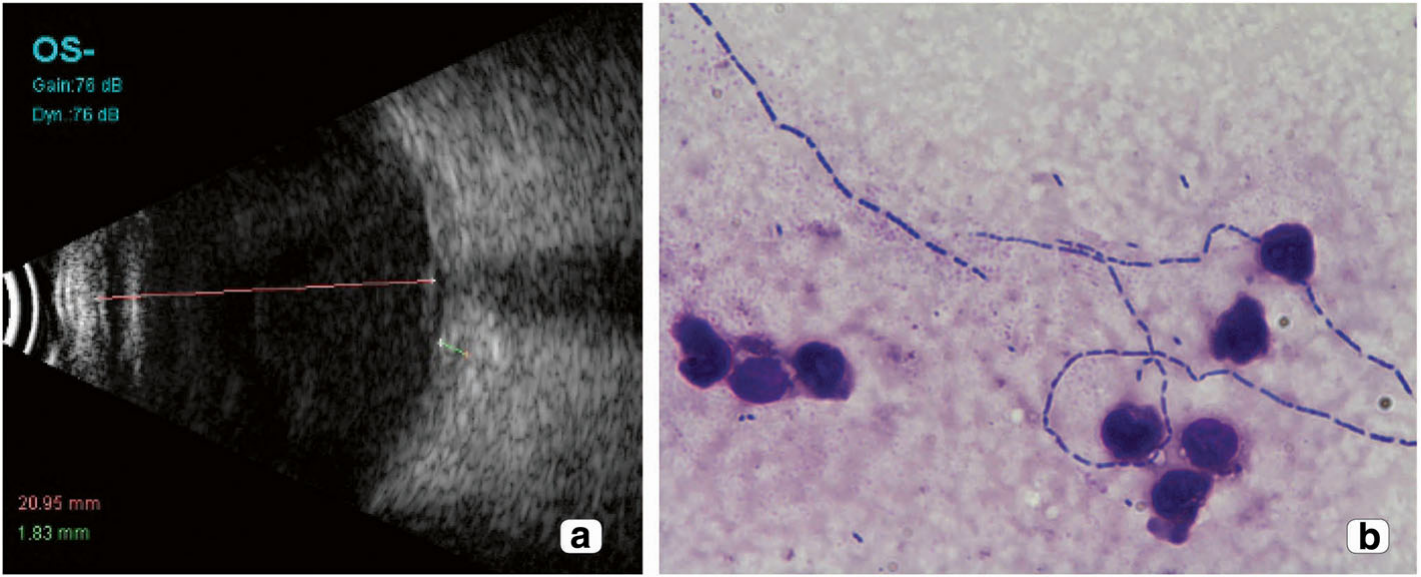
Case 3: **a** B-scan ultrasonography showing multiple hyperechoic areas with attached retina. **b** Giemsa stain of the vitreous biopsy showing filamentous bacilli (× 100) identified as *Pseudomonas aeruginosa* on culture

A 71-year-old diabetic and hypertensive male presented with decreased vision in the left eye since 2 days. The patient underwent a cataract surgery 15 days back elsewhere. On examination, the vision recorded in the right eye was 6/6 N6 and left eye was hand motions close to face. The left eye showed corneal edema, hypopyon, and circumcorneal congestion. A B-scan ultrasound showed moderate intensity echoes in the vitreous cavity with attached retina (Fig. 3a). A provisional diagnosis of post surgical endophthalmitis was made in the left eye, and the patient underwent a pars plana vitrectomy with intraocular antibiotic injections. The samples were sent to the microbiology laboratory the next day, and Gram and Giemsa stain of the vitreous biopsy then showed slender filamentous bacilli (B) resembling actinomycetes but were non-acid fast. Two days later, the culture showed presence of gray moist colonies, which was identified as *Morganella morganii*. The organism was resistant to tobramycin, tigecycline, chloramphenicol, ceftazidime, ciprofloxacin, moxifloxacin, imipenum, and colistin and was susceptible to amikacin, meropenum, piperacillin/tazobactum, and gentamicin (Table 1). Over the next 3 days, the patient underwent multiple intraocular antibiotic injections of vancomycin and ceftazidime. At the last visit, the vision recorded in the left eye was hand motions close to face with an attached retina on B-scan and reducing echoes.

## Discussion

Microscopy remains the cornerstone of the laboratory diagnosis of ocular infections, to guide further therapy. Bacteria can evolve remarkably quickly to be resistant to antibiotics under certain conditions, and filamentation is probably the first step in the evolution of resistance to mutagenic antibiotic exposure. Even in the hands of well-trained microbiologists and technologists, diagnosis may be hampered by a variety of changes in bacterial morphology including elongation, swelling, and filamentation. Bacteria can evolve remarkably quickly to be resistant to antibiotics under certain conditions, and filamentation is probably the first step in the evolution of resistance [6, 8]. Many microorganisms adopt a filamentous shape caused by cell-division arrest yet continue cell-volume growth in response to a variety of stressful environments, including nutrient deficiency, extensive DNA damage through the SOS response pathway, host innate immune responses [8, 9], desiccation, high pressure, and antibiotic treatment [10]. The filamentous cells can reach lengths of at least 50 times an ordinary bacillus and often grow much longer, thus resembling fungal hyphae [6]. Whereas true hyphae and bacterial filaments may appear similar, the Gram stain should help distinguish between these two entities. Fungal hyphae are always broader than 2 μm, may stain Gram positive, and will often show various degrees of branching and septation. Bacterial filaments appear more slender and may intertwine but do not demonstrate true branching.

In the three cases presented here, the filamentous organisms were initially misinterpreted as actinomycetes owing to the length and suspected branching, and only after review of culture identification the following day was it realized that the Gram-negative staining, along with the patient’s recent antibiotic exposure, could explain altered bacterial morphology. No actinomycete was grown in culture. Correlating the findings of a negative acid-fast stain with the results of a Gram stain is recommended to aid in making a diagnosis of the involvement of actinomycetes. We would also like to highlight the role of culture in identifying the causative organism in our cases which is why culture methods are still considered the “gold standard” in the diagnosis of endophthalmitis. However, in the case of a negative culture report, the organism would have been presumed to be an actinomycete or nocardia and intravitreal injection of amikacin (400 g in 0.1 ml), instead of ceftazidime (2.25 mg in 0.1 ml), would have been the drug of choice in the treatment along with systemic trimethoprim (160 mg) sulphamethoxazole (800 mg) combination. In addition to antibiotic treatment, some authors [11] recommend early surgical intervention (pars plana vitrectomy instead of vitreous biopsy) as they believe that inadequate response of actinomycetes to medical therapy is due to poor antibiotic penetration [11, 12]. Hence, to avoid misdiagnosis in such culture-negative cases, Gram stain results should be considered along with molecular testing of the sample like PCR and sequencing to aid in the diagnosis [13]. Additionally, when considered with relevant clinical information and correlation, the morphological features seen in biopsies may provide sufficient information to correctly identify a particular type of organism, as in our cases where all of them had a short history with acute presentation. In our study, all three filamentous bacteria were gram-negative bacteria which usually produce severe endophthalmitis with a poor chance of visual recovery. Additionally, two of the three cases were multi-drug resistant and thus the visual outcomes observed in our cases were not surprising.

## Conclusions

In conclusion, the filamentous growth of bacteria after the administration of sub-inhibitory concentrations of antibiotics or delay in processing may occur in vitreous specimens as a survival strategy. Thus, the possibility of drug resistance in these organisms should also be kept in mind when treating such cases. Features that can be used to distinguish bacterial filaments from actinomycetes are smaller size, absence of branching, and Gram-negative staining characteristics. Understanding the variations in bacterial morphology enables the clinical microbiologist and pathologists to communicate effectively, hopefully leading to an earlier, more precise diagnosis of patient disease.

## Abbreviation

CDVA: Corrected distance visual acuity
